# Ataxia and mobility in children following surgical resection of posterior fossa tumour: A longitudinal cohort study

**DOI:** 10.1007/s00381-021-05246-0

**Published:** 2021-07-07

**Authors:** H Hartley, S Lane, B Pizer, L Bunn, B Carter, E Cassidy, C Mallucci, R Kumar

**Affiliations:** 1 Alder Hey Children’s NHS Foundation Trust, Liverpool, UK; 2 University of Liverpool, Liverpool, UK; 3 University of Plymouth, Plymouth, UK; 4 Edge Hill University, Ormskirk, UK; 5 Freelance Academic, London, UK

**Keywords:** Paediatrics, Cerebellum, Rehabilitation

## Abstract

**Purpose:**

To report the course of ataxia in children up to 2 years post-operatively, following surgical resection of a posterior fossa tumour (PFT).

**Methods:**

Thirty-five children, (median age 9 years, range 4–15) having resection of PFT, were assessed using the Scale for the Assessment and Rating of Ataxia (SARA), Brief Ataxia Rating Scale (BARS) and the mobility domain of the Paediatric Evaluation of Disability Index (PEDI-m) at initial post-operative period (baseline), 3 months, 1 year and 2 years post-operatively.

**Results:**

Baseline median scores of the SARA and BARS were 8.5 (range 0–35.5), and 7 (0–25) respectively. Ataxia improved at 3 months (median SARA and BARS reduction 3.5 and 4, respectively). Additional gradual improvements in SARA were recorded at 1 (median reduction 2) and 2 years post-operatively (median reduction 0.5). Median baseline PEDI-m was 54.75 (range 15.2–100) with improvement at 3 months (median increase 36.95) and small improvement at 1 year (median increase 2.5) and 2 years (median increase 5.8). Children with medulloblastoma and midline tumours (median baseline SARA 10 and 11, respectively) demonstrated more severe ataxia than children with low-grade gliomas and unilateral tumours (median baseline SARA 7.5 and 6.5, respectively).

**Conclusion:**

The largest improvement in ataxia scores and functional mobility scores is demonstrated within the first 3 months post-operatively, but ongoing gradual improvement is observed at 2 years. Children with medulloblastoma and midline tumour demonstrated higher ataxia scores long term.

**Supplementary Information:**

The online version contains supplementary material available at 10.1007/s00381-021-05246-0.

## Introduction

Posterior fossa tumours (PFT) account for approximately 50% of all childhood brain tumours [1]. Management of PFT typically involves surgical resection, solely or in combination with adjuvant treatments such as radiotherapy and chemotherapy [2].

Ataxia is the predominant motor problem in children with PFT. Ataxia describes a number of related impairments including limb incoordination, tremor, gait disturbance, impaired balance and oculomotor and oromotor dysfunction [3]. Approximately 60% of children with PFT demonstrate ataxia pre-operatively [4]. The severity and incidence of ataxia may increase post-operatively [5].

Evolving evidence indicates that ataxia can persist in the long term in children with PFT. Robertson et al. [5] presented initial work in this area observing that children with severe ataxia at 1 month post-operatively continued to have ataxia at 1 year after surgery. Sønderkær et al. [6] reported that ataxia may persist at 10 years after surgery. More recent reporting with standardised outcome measures has also observed approximately 70% [7, 8] of children with PFT which demonstrate balance dysfunction more than 1 year following surgery.

Risk factors for ataxia are less well described than for other toxicity such as cerebellar mutism syndrome, where invasion of the brainstem is known to be a risk factor [5], and potential for reduction in risk of ataxia has not been widely explored. Kuper et al. [9] presented one of the few studies with longitudinal data, describing the recovery of 12 children with cerebellar tumour from the early post-operative period to reassessment at 3 months and 1 year. They noted ongoing recovery throughout the first year, although children with injury to the deep cerebellar nuclei had persistent impairment. These findings support the earlier cross-sectional study by Konzcak et al. [10].

In this paper, we present a new insight into the longitudinal course of ataxia and functional mobility using standardised measures in children with PFT from the peri-operative period, initial post-operative period and up to 2 years post-operatively. Additionally, we explore the impact of tumour location, histology and adjuvant treatment on severity of ataxia.

## Methods

### Design

Cohort prospective single site longitudinal study.

### Participants

Children who had surgical resection of a PFT and were aged between 4 and 18 years (inclusive) were eligible for inclusion. Due to the need to be able to follow the instructions required for assessment of ataxia, the lower age limit was set as 4 years old.

### Procedures

Children were recruited from a single tertiary neurosurgical/oncology unit in the UK between 2012 and 2018. The study was approved by the relevant local ethics and research and development committee (IRAS ID98449).

Potential participants were identified and screened for eligibility by the neurosciences therapy team. Informed and written consent was obtained from all children and/or their parents (with child assent).

Children were assessed initially as an inpatient within 1 week of surgical resection (baseline) and then in an outpatient clinic setting at 3 months, 1 year and 2 years post-operatively. Assessments were completed by a neuro-physiotherapist trained in the use of the standardised outcome measures. A subset of participants was also assessed pre-operatively; this occurred if children were stable pre-operatively and where timing of admission and surgery allowed for pre-operative assessment.

### Ataxia outcome measures

Children were assessed using the Scale for the Assessment and Rating of Ataxia (SARA scale) [11] and the Brief Ataxia Rating Scale (BARS scale) [12]. The SARA and the BARS are reliable and valid measures of ataxia for children with PFT [13]. The SARA is used in clinical paediatrics and has age-based normative reference values [14]. It has 8 items and has a total score out of 40, with a higher score indicating more severe ataxia. Three items of the SARA (gait, stance and sitting, total 18) have been deemed the Bal-SARA [15] and particularly represent balance impairment. The BARS has 5 items and is scored out of 30; a higher score indicates more severe ataxia. Both scales are quick and easy to use based on a standard clinical examination of ataxia.

The mobility domain of the Paediatric Evaluation of Disability Index (PEDI) was also used as a functional mobility measure [16]. The PEDI is a valid and reliable functional measure in children with acquired brain injury (validated from 6 months to 7 years, although it can be used in older children with functional difficulties) [16]. It has self-care, mobility and social function sections which can be used as a whole or as stand-alone domains. It is completed by the therapist or by parental questioning. Only the mobility domain of the PEDI (PEDI-m) was collected to minimise time burden of assessment for the participants. A higher PEDI-m score (range 0 to 100) represents a better level of physical function and correlates with lower overall measures of disability.

### Baseline disease descriptors

Baseline potential predictive factors of severity of ataxia were collected including tumour histology, age at diagnosis, adjuvant treatment (radiotherapy or chemotherapy), tumour location and surgical approach.

### Data analysis

The study was not designed as a comparative study; therefore, the data were summarised using descriptive statistics (including age at diagnosis, tumour location, tumour histology and adjuvant treatment). Medians and interquartile ranges were used to summarise continuous data because of the small number of observations. For categorical data, counts are reported. The SARA and the BARS demonstrated similar patterns, and as the SARA is more widely used in clinical paediatrics, further analysis regarding ataxia was undertaken using the SARA only.

Patterns of change in outcome measures were therefore explored graphically (using Box-Whisker plots and time series plots) for the SARA and the PEDI-m. Participants were stratified by putative predictive factors of tumour histology (medulloblastoma versus low-grade glioma), tumour location and adjuvant treatment The PEDI is normative-referenced between 6 months and 7.5 years. In line with recommendations, raw scores were converted to scaled scores to enable the use of the PEDI-m in children over the age of 7.5 years.

## Results

### Study sample

Thirty-five participants were recruited to the study. Participant characteristics are detailed in Table 1.

**Table 1:** Participant characteristics at baseline (*n* = 35)

Characteristic	Value
Gender (*n*)	
Male	21
Female	14
Age at time of recruitment (years)	
Median (range)	9 (4-15)
Histology (*n*)	
Low-grade glioma	17
Medulloblastoma	11
Ependymoma	4
Other (e.g. schwannoma)	3
Location (*n*)	
Midline	23
Unilateral	12
Adjuvant treatment (*n*)	
Surgery	19
Surgery and radiotherapy	5
Surgery and radiotherapy and chemotherapy	11

### Ataxia outcome measures

Descriptive analysis of the outcome measures is presented in Table 2. The SARA range at baseline was 0 to 35.5. The BARS range at baseline was 0 to 25. A higher SARA (out of 40) and BARS score (out of 30) represents more severe ataxia. The SARA and BARS scores were in agreement demonstrating a similar trend and therefore for the rest of the results as detailed in the data analysis plan, only the SARA is presented alongside the PEDI-m. The PEDI-m range at baseline was 15.2–100 (higher score representing better functional mobility, maximum score 100). A ceiling effect of the PEDI-m is noted with 39% of children reaching the maximum score of 100 at 1 year post-operatively

**Table 2:** Participant ataxia and mobility scores

	Pre-operative score *n* = 22	Baseline *n* = 35	3 months *n* = 31(*n* = 4 missing)	1 year *n* = 33 (*n* = 1 missing, *n* = 1 died)	2 year *n* = 31 (*n* = 2 missing, *n* = 2 died)
SARA					
Group median (IQR)	6 (8.5)	8.5 (9.5)	5 (4.5)	3 (6)	2.5 (5)
range	(0–24)	0–35.5	0–30	0–28.5	0–16.5
Bal-SARA					
Group median (IQR)	3 (5.25)	5 (5)	2 (2)	1 (3)	1 (3)
range	0–16	0–18	0–17	0–16	0–8
BARS					
Group median (IQR)		7 (10.5)	3 (3.25)	3 (5)	2 (5)
Range		0–25	0–24	0–24	0–16
PEDI-m					
Group median (IQR)		54.75 (30.9)	91.7 (20.2)	94.2 (20.2)	100 (16.15)
range		15.2–100	20.9–100	30.6–100	47–100

Twenty-two children were also assessed pre-operatively using the SARA scale. There was an initial increase in SARA post-operatively (worsening ataxia) followed by improvement on average for the group. The ataxia scores at 3 months post-operatively are lower (better) than the pre-operative assessment scores, indicating improvement in ataxia 3 months after surgery. At 2 years post-operatively, ataxia severity had reduced by half of the pre-operative level (median values for the overall sub-group).

The change in Bal-SARA represents a large proportion of the initial change in SARA score. The Bal-SARA includes gait, stance and sitting items of the SARA scale.

### Effect of tumour location

**Fig. 1:**
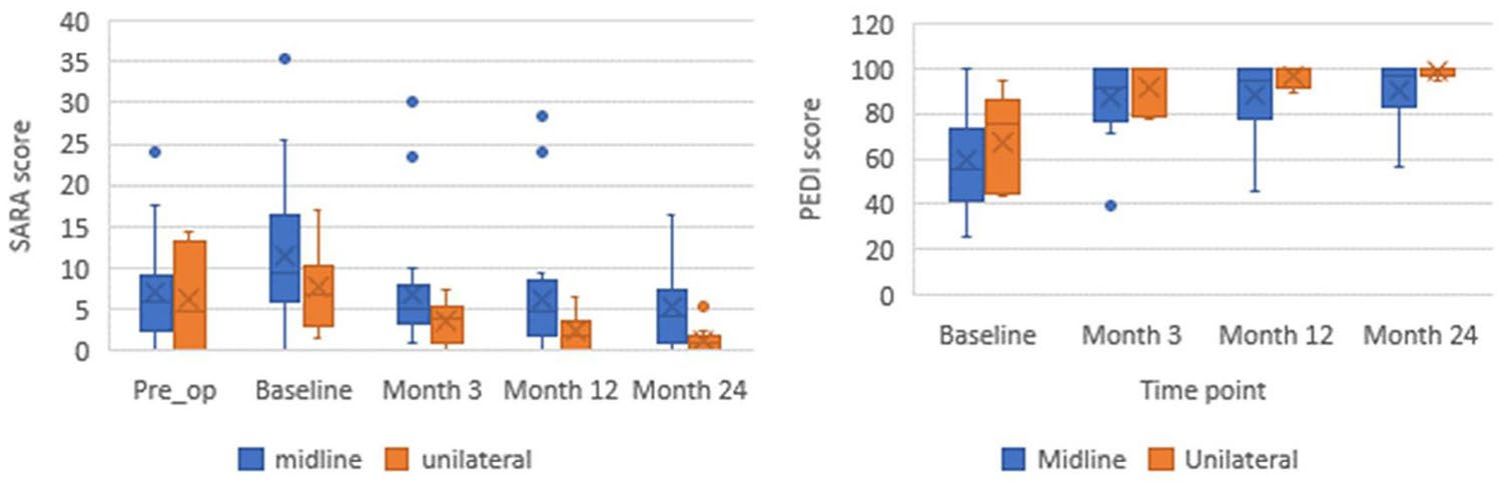
Group median SARA and PEDI-m scores for children (*n* = 35), dependent upon tumour location. All available scores plotted, n=23 midline, n=12 unilateral. Pre op SARA available for n=22

**Fig. 2:**
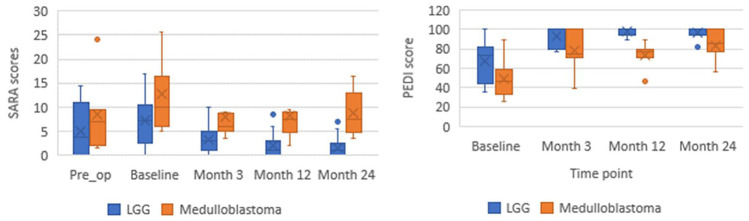
Group median scores of SARA and PEDI-m scores for children (*n* = 28), dependent upon tumour histology. All available scores plotted, n=17 LGG, n=11 medulloblastoma. Pre op SARA for n=19

**Fig. 3:**
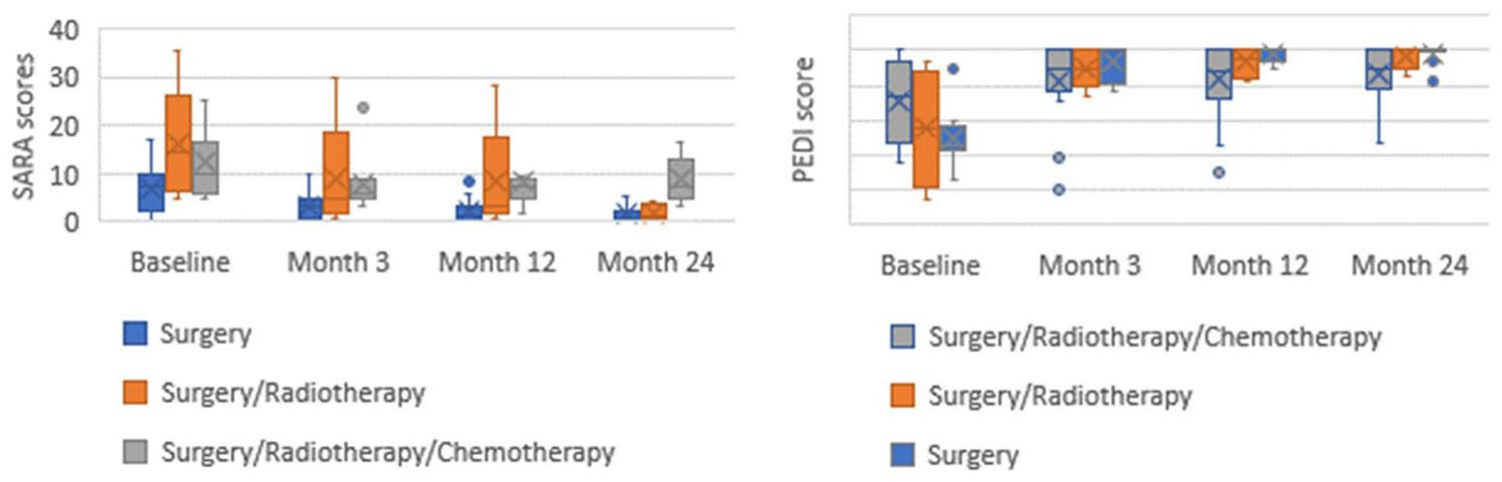
Group median scores of SARA and PEDI-m scores for children (*n* = 35), dependent upon adjuvant treatment

Further analysis is completed to compare change over time for outcome measures dependent on tumour location (Fig. 1). Alternative graphical representation of line graphs for Figs. 1, 2 and 3 are available in supplementary material (Online Resource 1).

A similar trajectory for both tumour locations was noted; however, children with unilateral tumours demonstrated lower SARA scores than those with midline tumours, indicating less ataxia throughout all time points. The change in PEDI-m scores demonstrated the same trajectory as previously noted, with rapid increase in scores between baseline and 3 months, although there is less difference in scores between tumour location. The ceiling effect of the PEDI-m is observed.

Further analysis of ataxia considering a more detailed breakdown of tumour location (4th ventricle, 4th ventricle/cerebello-pontine (CP) angle, CP angle, cerebellar hemisphere and brain stem) is detailed in supplementary material (Online Resource 2). No firm conclusions can be made due to small numbers in each group; however, this classification may be of value in a larger population to identify further the effect of tumour location.

### Effect of tumour histology

Further analysis was completed to determine whether changes in outcome measure score were dependent on tumour histology. The two most common tumour histologies, medulloblastoma (*n* = 11) versus low-grade glioma (*n* = 17), are presented for all assessment timepoints.

Although both tumour types follow a similar trajectory, i.e. rapid drop in ataxia from baseline to 3 months post-operatively, children with medulloblastoma had higher SARA scores (more severe ataxia) and lower PEDI-m scores (more mobility function impairment) than children with low-grade gliomas (Fig. 2). The gap in ataxia scores appears to widen after 1 year; children with medulloblastoma had higher ataxia severity at 2 years post-operatively. It is noted that the gap between the tumour histology groups is wider than the graphs comparing tumour location.

Both the SARA and PEDI-m appear to be able to distinguish between the two tumour types. Again, there is a ceiling effect for the PEDI-m.

### Effect of adjuvant treatment

Further analysis was completed to determine whether changes in outcome measure were dependent on adjuvant treatment. Surgery only (*n* = 19) versus surgery and radiotherapy (*n* = 5) versus surgery, radiotherapy and chemotherapy (*n* = 11) are presented.

A reduction in ataxia is observed for all three groups. However, following the 3-month assessment children who had received surgery, radiotherapy and chemotherapy demonstrated sustained higher ataxia scores up to 2 years post-operatively. It should be noted that only 5 children are in the surgery and radiotherapy group.

### Effect of surgical approach

Further analysis was completed to determine whether changes in outcome measure were dependent on surgical approach. This is presented in supplementary material (Online Resource 2).

### Individual change scores

There was a large range in individual change scores highlighting individual variability. Individual change scores are presented further in supplementary material (Online Resource 2).

### Risk factors for persistent ataxia

Further analysis regarding risk factors for persistent ataxia (dentate nucleus invasion, cerebellar peduncle involvement, brain stem invasion and presence of hydrocephalus) is presented in supplementary material (Online Resource 2). Analysis was carried out to determine if the presence of these factors raised the risk of moderate/severe ataxia (defined as SARA score greater than 7, [13]) at 12 months post-operatively. No clear risk factors are identified, although caution should be applied in interpreting these results due to low numbers in each group.

## Discussion

This is the largest published longitudinal study to focus specifically on ataxia in children with PFT and the first to report the course of ataxia up to 2 years after surgery including pre- and post-operative scores. Putative factors (tumour histology, location and adjuvant treatment) that may predict the severity and course of long-term ataxia were also identified.

The most rapid improvement in ataxia was noted in the first three post-operative months. Much smaller change was noted thereafter up to 2 years post-operatively. Although the minimally clinically important difference (MCID) for the SARA in children has not been determined, the MCID for adults has been reported as 1.1 [17]. The MCID might be higher in children with increased variability in performance (particularly in younger children); however, the group median change was more than three times the adult suggested value (3.5 between baseline and three months post-operatively), which highlights the potential clinical significance of this change. A threshold of change of 2.0 or more in SARA has been suggested to allow for physiological fluctuation in healthy children [14]. The change seen in this study is higher than this. The Bal-SARA subset appears to account for a large proportion of this change, indicating that balance in particular rather than limb coordination improves in the initial post-operative period.

For the PEDI-m, a rapid improvement in mobility was also noted at the 3-month assessment (exceeding the MCID suggested by Iyer et al. [18] of 11%), followed by minimal change after this point, although a ceiling effect for the PEDI-m was noted in this population (13 of the children scored a maximum of 100 at 1 year post-operatively). This means ongoing higher level mobility problems may not be detected by this tool. Caregiver assistance scores for the PEDI could be used in future studies as this might highlight more subtle change, such as the child being able to do a task with less assistance, which might have a meaningful impact on family life. Future research might usefully consider alternative outcome measures examining balance impairment such as the paediatric balance scale [19] and measures of participation.

Analysis of a subset of children who were assessed pre-operatively demonstrated an initial increase in ataxia post-operatively, followed by a drop below the pre-operative level by the 3-month assessment time point. Although it is known that approximately 60% of children will present with ataxia pre-operatively [4], this is the first time that the change during this time period has been quantified, and it would be of value to confirm these findings with a larger sample.

SARA scores are age-dependent in healthy children [14, 20]. However, due to the small number of participants in this study, it was not possible to consider age formally as a confounding factor in the analysis. Although children may not reach the adult optimum for the SARA (and BARS) scores until the age of 10, Lawerman et al. [14] suggested that the median total SARA score is under 1.5 in healthy children from age 6 to 10 years. Additionally, between each age year increment, only a small decrease of 0.5–1 is expected on the SARA (e.g. median SARA score in healthy 6 year olds is 1.5, in 7 year olds, 0.5). This would suggest that the large changes seen in the first 3 months following surgery are consistent with recovery and repair of brain function and not just age-related maturational changes which occur over a longer time course.

The pattern of change reported in this study is supported by results from a longitudinal study which measured function in 12 children with PFT (age 6–17 years) who were followed up to 1 year postoperatively [9]. A reduction in ataxia score was observed (using the International Cooperative Ataxia Rating Scale ICARS) from the acute post-operative period to 1 year after surgery, with a statistically significant reduction in the first 3 months, but not from 3 months to 1 year, similar to findings from our study. Kuper et al. [9] observed an improvement between 3 months and 1 year after surgery in upper limb motor function and body sway measured using instrumented motion analysis.

Kuper et al. [9] suggested that reduction in oedema affecting the deep cerebellar nuclei was a predictor of early functional recovery. If the resolution of oedema happens over days to weeks, an additional assessment time point at 1 month could demonstrate if the time of most rapid change (improvement) corresponds with the expected resolution of oedema. If this is not demonstrated, other mechanisms such as axonal sprouting and long-term potentiation would need to be considered to explain recovery in the first three months (acute phase). The recovery trajectory identified in the present study is consistent with studies of children with acquired brain injury (from other pathologies) which also indicate that the most rapid change occurs in the first 6-month post neurological insult [21]. Further examination of potential risk factors for persistent ataxia, e.g. dentate nucleus invasion, cerebellar peduncle involvement, brainstem invasion and hydrocephalus, would be of value with a larger population to provide further prognostic information.

Children with medulloblastoma and those with midline tumours demonstrated higher ataxia scores (and lower functional mobility scores) throughout the trajectory of their recovery. This is in line with other studies reporting that children with medulloblastoma have a higher incidence of ataxia and cerebellar mutism syndrome than children with other tumour types [5, 22–25]. In contrast, children with low-grade gliomas (LGG) (predominantly pilocytic astrocytomas) typically demonstrated lower ataxia scores. These findings are further supported by research that reports children with LGG most often have mild cerebellar dysfunction [26].

It is difficult to determine the influence of tumour histology compared with tumour location on ataxia severity, as there is an inherent link between the two factors: medulloblastoma more often being midline than lateralised in the cerebellum and LGG showing the converse relationship. Lesions to the deep cerebellar nuclei and inferior vermis, which are structures at or close to the midline, and the involvement of the efferent cerebello-thalamo-cerebral tract have been linked with persistent motor deficit in children [10, 24, 27]. Further detailed analysis regarding tumour location has been proposed in this study; however, due to small numbers, no conclusions were able to be made at this time. Future research should consider utilising detailed imaging of specific tumour location, cerebellar and tumour volume as predictors of severity of ataxia. Additionally, no clear observations were able to be made about the impact of surgical approach on ataxia in this study; however, this may be of interest with a larger population.

Findings from this study show a wider gap in ataxia scores depending on tumour histology than observed due to tumour location, suggesting that histology differentiates the strata of participants better than tumour location. Despite not being independent factors, this indicates that anatomical predilection of tumour histology is not the whole answer, and other elements should be considered, the most obvious being the need for adjuvant oncology treatment, i.e. radiotherapy and chemotherapy for children with medulloblastomas. The results showed that children who had received both chemotherapy and radiotherapy demonstrated a sustained higher level of ataxia which is in keeping with previous research highlighting the impact of chemotherapy in addition to surgery and radiotherapy on motor function [7]. This may explain the increase in SARA score for children with medulloblastoma after 3 months as typically, a 6-week course of radiotherapy is commenced within 1 month of surgery, and maintenance chemotherapy begins 6 weeks following this. Peripheral neuropathy which is a known side effect of chemotherapy can impair postural control strategies and result in functional balance problems [28].

Overall, tumour location, histology and adjuvant treatment are all interlinked, e.g. children with medulloblastoma because of the nature of the tumour type typically feature midline tumours and receive multimodal therapy (i.e. surgery, radiotherapy and chemotherapy). Therefore, in the future, a larger study which includes multivariate analysis would be of benefit to identify the impact of single variables to determine if any particular factor raises the risk of ataxia severity.

The small number of children with severe ataxia (SARA greater than 14 [13]) in this study meant it was not possible to compare the pattern of change across the trajectories of children with severe ataxia with those with mild/moderate ataxia. This analysis should be undertaken in future research to determine if there is any difference in recovery. Mapping individual trajectories (as in supplementary material, Online Resource 2) with a larger dataset might also be of value to identify characteristics of participants who show potential for ongoing improvement and to help focus rehabilitation interventions. Identifying children who have less potential to improve would also be of value in counselling families. It is acknowledged that during their initial inpatient stay, children had access to daily rehabilitation by the same therapy team; however, following discharge, rehabilitation intensity varied according to the child’s need and the availability of local services. Therefore, reviewing intensity of rehabilitation alongside trajectory of change may also be of interest.

This is the largest study to date reporting longitudinal ataxia severity in children with PFT. The strengths of this study include a prospective design using standardised validated outcome measures [13] over a 2-year period. The number of participants remains relatively small particularly for subgroup analysis. A further limitation is that the children were recruited from one tertiary site.

Our study provides information about the long-term course of ataxia in children following surgical resection for PFT. Children often have worse ataxia after surgery, before improving to better than they were prior to surgery. Children with medulloblastoma and midline tumours have more long-term balance and coordination problems compared to children with LGG or unilateral tumours. The most rapid improvement was seen in the first 3 months postoperatively followed by a small change up to 2 years post-operatively. Any late changes do not appear to be explained simply by developmental maturation of cerebellar function especially in adolescents, though the impact of age should be examined further.

Although the findings would benefit from further confirmation with a larger dataset, they demonstrate the need to examine the potential of targeted rehabilitation interventions more than 1 year post-operatively when some children otherwise typically appear to demonstrate minimal change.

## Supplementary Information

ESM 1 (DOCX 88 kb)

ESM 2 (DOCX 59 kb)

## Data Availability

The datasets generated during and/or analysed during this study are available from the corresponding author on reasonable request.

## Declarations

### Disclaimer

The views expressed are those of the authors and not necessarily those of the NHS, the NIHR, or the Department of Health and Social Care.
