# Supplementary material for: Ataxia and mobility in children following surgical resection of posterior fossa tumour: A longitudinal cohort study
Source: Childs Nerv Syst. 2021 Jul 7;37(9):2831–8. doi: 10.1007/s00381-021-05246-0 (PMC8423635; doi:10.1007/s00381-021-05246-0)
Supplement: Supplementary file 1 — (DOCX 88 kb) [file 381_2021_5246_MOESM1_ESM.docx]

**Online Resource 1 - Supplementary Material (Alternative line graphs for Figures 1-3.)**

**Fig. 1** Group median SARA and PEDI-m scores for children (n=35), dependent upon tumour location

Pre op SARA available for n=22

**Fig. 2** Group Median scores of SARA and PEDI-m scores for children (n=28), dependent upon tumour histology

Pre op SARA available for n=19

**Fig. 3** Group median SARA and PEDI-m scores for children (n=35), dependent upon adjuvant treatment
