# Supplementary material for: Ataxia and mobility in children following surgical resection of posterior fossa tumour: A longitudinal cohort study
Source: Childs Nerv Syst. 2021 Jul 7;37(9):2831–8. doi: 10.1007/s00381-021-05246-0 (PMC8423635; doi:10.1007/s00381-021-05246-0)
Supplement: Supplementary file 2 — (DOCX 59 kb) [file 381_2021_5246_MOESM2_ESM.docx]

***Online Resource 2 - Supplementary Material***

*Detailed tumour location*

Further analysis was completed using a more detailed tumour location, classified as, 4^th^ ventricle (n=4), 4^th^ ventricle/CP angle (n=8), CP angle (n=4), cerebellar hemisphere (n=13) and brain stem (n=5). Typically, ataxia scores are higher for children with 4^th^ ventricular tumours and lower for children with hemispheric tumours, however, no firm conclusions can be drawn in view of low numbers in each group.

This data is presented graphically below in Supp. Fig1.


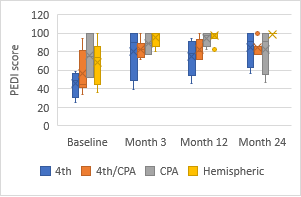
**Supp. Fig 1.**  Group Median scores of SARA and PEDI-m scores dependent upon detailed tumour location
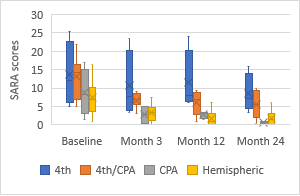


*brainstem location not represented graphically due to low numbers/missing data.

*Surgical Approach*

Further analysis was completed using surgical approach, classified as, midline direct (n=3), midline/retrosigmoid (n=4), retrosigmoid (n=5), supracerebellar (n=1), telovelar (n=7), transvermian (n=3), transcerebellar (n=11). No clear differences are observed between surgical approach, however, caution should be applied in interpreting the results due to low numbers in each group. The three largest groups are illustrated graphically below in Figure Supp. Fig. 2.

**Supp. Fig. 2** Group Median scores of SARA and PEDI-m scores dependent upon surgical approach
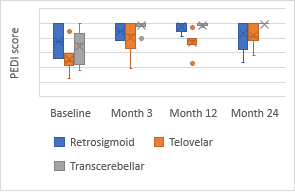

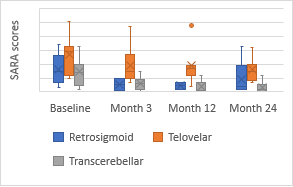


*Individual Change Scores*

Considering individual change scores per participant there was an initial worsening of ataxia for most participants, from pre to post-operatively with a median change in SARA of +3.5, with a large range of change from -18 to +18.5. Post-operatively from baseline to three months following surgery there was a median change of -4.5 for the SARA (improvement). The range of change for individual participants was -16.5 to +2. Additional gradual improvements for SARA individual change scores were recorded at one year with a median change of -1 (range -4.5 to +2.5) and two years with a median change of -0.5 (range -4.5 to +13.5). Return of functional mobility behaved similarly, with a rapid increase in individual change scores, median change +27.8, (range -9 to +64.1) from baseline to three months post-operatively, indicating an improvement in mobility, with a plateau at one and two years post operatively, median individual change score of 0.

The large range in individual change scores highlights individual variability. This is illustrated in Supp. Fig. 3 where three participants with different individual trajectories of ataxia are presented, one participant demonstrated a typical course, one participant demonstrated more severe ataxia with later improvement and one participant demonstrated minimal ataxia with consistently low scores.

**Supp. Fig 3.** Example of individual participant trajectory of ataxia (for 3 participants)

*Risk factors for persistent ataxia*

Table Supp. Table 1 illustrates analysis of presence of risk factors in relation to risk of ataxia severity at 12 months post-operatively. No clear risk factors are identified however, caution should be applied due to low numbers in each group for analysis.

**Supp. Table 1**. Outcome SARA score > 7 at 12 months

| Variable | Mild Ataxia | Moderate/Severe Ataxia | Significance |
| --- | --- | --- | --- |
| N | 25 | 7 |  |
| Dentate nucleus invasion n (%). no  Yes | 7 (28.0)  18 (72.0) | 2 (28.6)  5 (71.4) | P > 0.99^1^ |
| Hydrocephalus n (%)  no  yes | 5 (25.0)  20 (75.0) | 0  7 (100.0) | P = 0.56^1^ |
| Cerebellar Peduncle involvement n (%)  no  yes | 12 (48.0)  13 (52.0) | 2 (28.6)  5 (71.4) | P = 0.43^1^ |
| Brain stem invasion n (%)  no  yes | 14 (56.0)  11 (44.0) | 3 (42.9)  4 (57.1) | P = 0.68^1^ |

1 Fisher’s exact test
